# Application of remote sensing techniques to deal with scale aspects of GRACE data to quantify groundwater levels

**DOI:** 10.1016/j.mex.2023.102108

**Published:** 2023-03-05

**Authors:** Durga Prasad Panday, Manish Kumar

**Affiliations:** a Sustainability Cluster, School of Engineering, University of Petroleum and Energy Studies, Dehradun, Uttarakhand, India; b Escuela de Ingeniería y Ciencias, Tecnologico de Monterrey, Campus Monterey, Monterrey 64849, Nuevo Leon, Mexico

**Keywords:** GRACE, Spatial variations, GLDAS, GRACE satellite data extraction and application

## Abstract

Groundwater has become an indispensable source of irrigation and drinking water. Industrial dependence on groundwater has also increased drastically. This has led to the rapid exploitation of groundwater. There is accelerating concern about the depletion of groundwater water levels and the deterioration of groundwater quality due to geogenic and anthropogenic causes. The availability of groundwater data is a huge concern, as it requires both time and capital. GRACE satellite project has become a very important tool for groundwater data access. The latest version of GRACE data provides terrestrial water storage, which is the sum of surface and groundwater. The present study details the method to access GRACE satellite data and prepare a spatial map for analysis. It also discusses how to handle data at different resolutions to quantify meaningful correlations. Further, groundwater data is correlated with nitrate data (both are at different grid resolutions) to throw light on the relationship between the important anthropogenic contaminant (nitrate) and groundwater levels. This provides insights into the linkage of quantity with quality. In brief, the important contributions of the paper are:

• To provide the methodology to access GRCAE data and prepare spatial maps.

• To handle the variables at different grid resolutions.

• To correlate two GIS maps at different spatial resolutions.

## Highlights

• GRACE satellite provides continuous terrestrial water storage data.
• GLDAS data along with GRACE provides global groundwater data at monthly scale.
• ArcMap can easily handle netcdf file and scale issues in data.
• Fishnet technique helps in harmonising the spatial resolutions of each variable.

## Graphical Abstract

**Figure:**
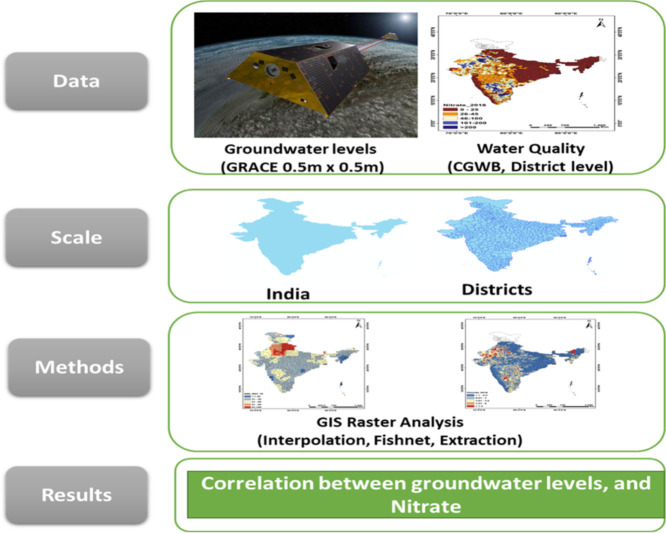

Specification Table

**Table:** 

Subject area:	Water Resources Engineering
More specific subject area:	Groundwater, Water Quality
Method name:	GRACE satellite data extraction and application
Name and reference of original method:	GRACE data extraction and analysis. Landerer, F. W., Flechtner, F. M., Save, H., Webb, F. H., Bandikova, T., Bertiger, W. I., et al. (2020). Extending the global mass change data record: GRACE Follow‐On instrument and science data performance. Geophysical Research Letters, 47, e2020GL088306. https://doi.org/10.1029/2020GL088306
Resource availability:	http://grace.jpl.nasa.gov/

## Introduction

Groundwater is an indispensable source for drinking, irrigation, and industrial requirement. This has led to the rapid exploitation of groundwater resources leading to severe deterioration of the groundwater quality and depletion in the levels as well [1]. Hence, quality and quantity are significantly compromised [5]. Climate change has further triggered an increase in hydroclimatic extremes frequency [2], [4]. The major issue regarding groundwater research is the accessibility of the data. Data collection requires an investment of time and capital. The government agencies in India like CGWB (Central Ground Water Board) and CPCB (Central Pollution Control Board) collect the data at the district level but the sampling frequency is less (3 times a year). They conduct pre-monsoon and post-monsoon sampling. The present GRACE mission provides continuous data (0.5m x 0.5m scale). Data is at the monthly scale and have a good temporal resolution.

With the rapid advancement in the field of remote sensing and increasing availability of satellite data in the open domain, researchers are correlating quantity with quality. The present work demonstrates the methods to access GRACE data (available for download in NetCDF file format) and convert it into GIS files (vector and raster) and the convenient excel format.

In brief, the important contributions of the paper are:

• To provide the methodology to access GRCAE data and prepare spatial maps
• To handle the variables at different grid resolutions.
• To correlate two GIS maps at different spatial resolutions.

## Materials and methods

**GRACE Data:** GRACE (0.5m x 0.5m) resolution data. GRACE satellite provides terrestrial water storage (TWS) relative to the time mean (2004-2009). Data retrieved from http://grace.jpl.nasa.gov/data/get-data/jpl_global_mascons/ employs a coastal resolution improvement (CRI) filter, which counters the errors due to signal leakages across coastlines. The present dataset is already smoothened and can be used directly to get TWS [3]. We have used data from the year 2015 to demonstrate the method.

**GLDAS Data:** GRACE data provides total water storage. To obtain the groundwater levels, we have subtracted the below-mentioned variables available under separate GLDAS data (1m x 1m). Since GLDAS data is available at a lower resolution, we have downscaled it using GIS software.

a) Plant canopy surface water (kgm^−2^)
b) Storm surface runoff (kgm^−2^)
c) Soil moisture content (0-10 cm underground) (kgm^−2^)
d) Soil moisture content (10-40 cm underground) (kgm^−2^)
e) Soil moisture content (40-100 cm underground) (kgm^−2^)

**Water Quality data:** We have targeted geogenic contaminant (Nitrate). The data is obtained from CGWB (http://cgwb.gov.in/wqreports.html) for the year 2015. CGWB conducts sampling in May and hence we have used GRACE data for May.

**Fig. 1:**
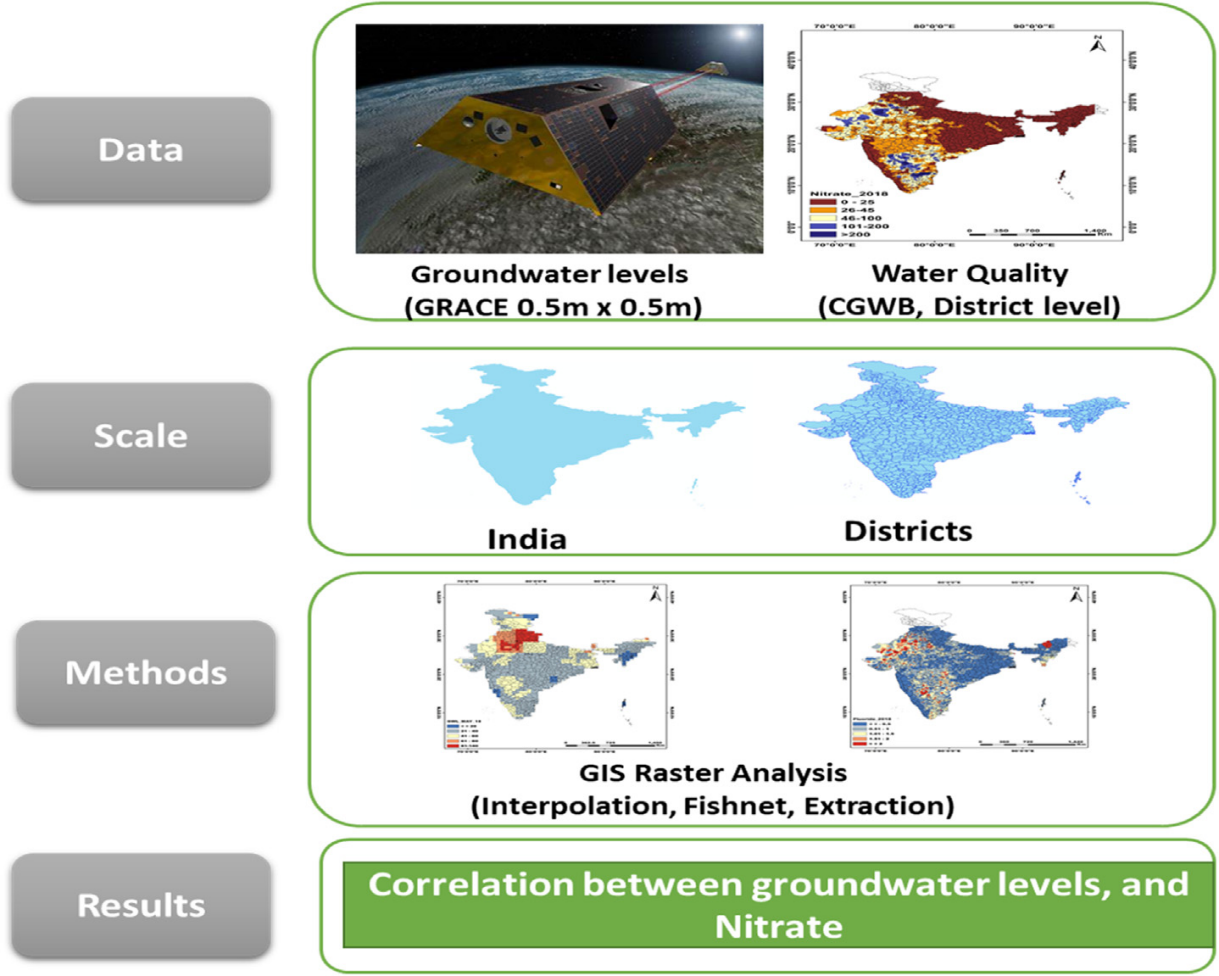
Flowchart illustrating the methodology for the development of the materials.
v. Since the resolutions of GRACE (TWS, 0.5m x 0.5m) data and GLDAS (0.25m x 0.25m) are different, they cannot be subtracted directly. Raster maps as shown in Figs. 2 and 3 can be prepared (explained in step 6) and then subtracted using the raster calculator tool in ArcMap.

**Fig. 2:**
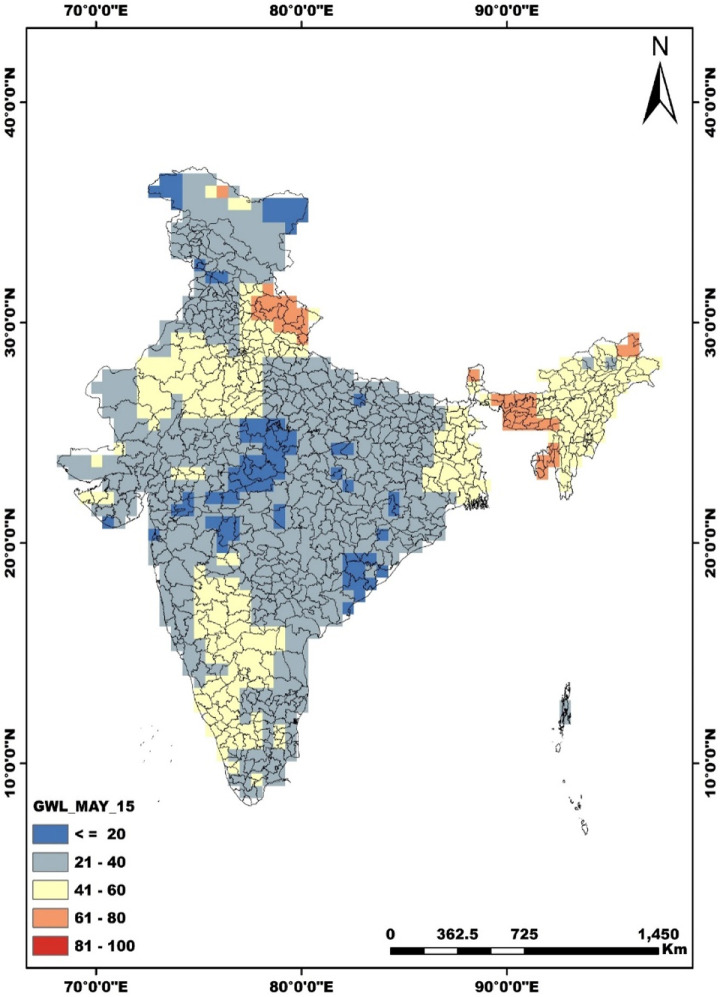
Spatial distribution of groundwater levels for India (cm) for May 2015. The map highlights various regions of groundwater levels decline relative the time mean of 2004-2009. This map is obtained after subtracting the GRACE terrestrial water storage raster map from the GLDAS data raster map.

**Steps for GRACE data extraction and analysis:**

i. GRACE and GLDAS data files are in the .netcdf format. It can be opened either through programming tools like python, R, MATLAB, etc., or through the GIS software.
ii. ArcGIS can easily access the .netcdf file format and convert it into the feature format (or tables format for analysis in excel). The .netcdf to feature tool in the multi-dimensional tools ArcMap (module of ArcGIS) can directly convert the .netcdf file into excel file format. The region of interest can then be clipped or narrowed down as per the user's requirements.
iii. GRACE satellite data provides terrestrial water storage, which is the sum of surface water (runoff), soil moisture at different depths, and groundwater.
iv. Hence, GRACE data is subtracted from the GLDAS data to finally obtain the groundwater levels data. All the GLDAS data variables have units of (kgm^−2^). Hence, it is converted to depth by dividing by 1000 (density of water 1000 kgm^−3^) and again multiplying by 100 to convert it into cm. The different GLDAS variables are added and the resultant variable is subtracted from the GRACE TWS (data provided in cm units) data to finally obtain the groundwater levels in cm (Figs. 1 and 2).

**Fig. 3:**
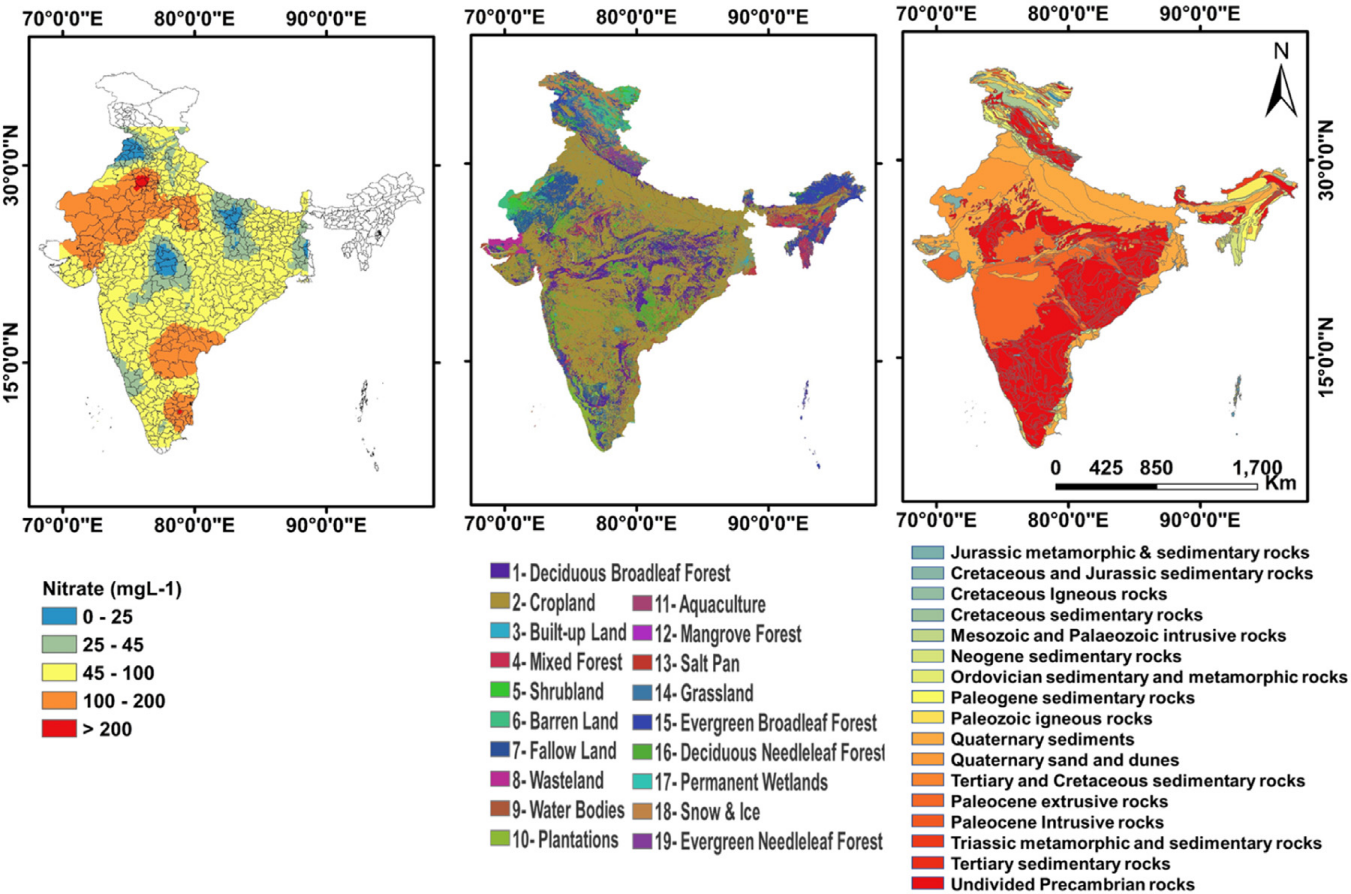
Spatial distribution of nitrate concentration for India (in mgL^−1^ for May 2015) (Left). Middle and right plots show land use and geological settings for India respectively. The permissible limit of nitrate as per BIS is mgL^−1^. (White color denotes that data is not available for the area).
vi. Raster map prepared using the spatial interpolation tool (available in spatial analyst tool in ArcMap). The options available are IDW (inverse distance weighting), Kriging, etc.
vii. Similarly, the spatial distribution map for the nitrate is prepared. Nitrate data provided by CGWB is available at the district scale. Hence, it cannot be directly compared with the groundwater level data.
viii. The fishnet option in the data management tool in ArcMap is very useful in this regard. After creating a fishnet, we can transfer the data of the groundwater levels and nitrate belonging to the same spatial location on the fishnet grid. It is done using the extract multiple values to grid points option in ArcMap.
ix. The fishnet file can be saved as .shp file and easily converted to excel for further analysis.
x. Finally, the correlation is calculated using python's seaborn library.

Fig. 3 highlights the spatial distribution of nitrate concentration based on [1] data. Dataset is divided in 5 categories. The last three categories (shown by yellow, orange and red) highlight the concentration above the prescribed BIS limit of 45 mgL^−1^. As clearly evident from the map, there are very few patches of blue color, signifying the area safe from nitrate contamination. There exists a weak correlation(r = -0.14) highlighting that groundwater level for 2015 is not the only influencing factor for nitrate water quality and investigating other important factors like land-use, geological settings, precipitation, hydrology and morphology, etc. are required. Fig. 3 also highlights geological and land use setting for India.

## CRediT authorship contribution statement

**Durga Prasad Panday:** Writing – original draft, Methodology, Visualization. **Manish Kumar:** Conceptualization, Visualization, Writing – review & editing, Supervision.

## Declaration of Competing Interest

The authors declare that they have no known competing financial interests or personal relationships that could have appeared to influence the work reported in this paper.

## Data Availability

Data will be made available on request.
